# Impact of recent guideline changes on aspirin prescribing after knee arthroplasty

**DOI:** 10.1186/s13018-016-0456-0

**Published:** 2016-10-20

**Authors:** Sarav S. Shah, Alexander M. Satin, James R. Mullen, Sara Merwin, Mark Goldin, Nicholas A. Sgaglione

**Affiliations:** 1 Department of Orthopaedic Surgery, Long Island Jewish Medical Center, 270-05 76th Ave, New Hyde Park, NY 11040 USA; 2 Department of Orthopaedic Surgery, Montefiore Medical Center, 111 E 210th St, Bronx, NY 10467 USA; 3 Department of Medicine, Long Island Jewish Medical Center, 270-05 76th Ave, New Hyde Park, NY 11040 USA

## Abstract

**Background:**

Prior to 2012, the American Academy of Orthopaedic Surgeons (AAOS) and American College of Chest Physicians (ACCP) differed in their recommendations for postoperative pharmacologic venous thromboembolism prophylaxis (VTEP) after total joint arthroplasty. More specifically, aspirin (ASA) monotherapy was not endorsed by the ACCP as an acceptable prophylaxis. In 2012, the ACCP supported ASA monotherapy compared with no prophylaxis. Our aim was to investigate the impact of the convergence of ACCP and AAOS recommendations on surgeon prescribing patterns after knee arthroplasty (KA).

**Methods:**

This is a retrospective chart review. We collected data to assess preoperative VTE risk and examined VTEP prescriptions on postoperative day 1 (POD1) and at discharge (D/C) from 7/2008 to 12/2011 (pre-period) and 1/2012 to 7/2014 (post-period). Adult patients undergoing primary and revision KA were identified by ICD-9 procedure codes. Patients on preoperative full-dose anticoagulation and with hypercoagulability disorders were excluded.

**Results:**

Of 368 records reviewed, 329 were included in the analysis. There were no differences between the two period groups for age, sex, BMI, estrogen therapy, malignancy, smoking status, prior VTE, bilateral procedures, or surgery within 3 months. On POD1, in the pre-period, 4.6 % were prescribed ASA monotherapy versus 44.4 % in the post-period (*p* < 0.001). On D/C, in the pre-period, 13.9 % were prescribed ASA versus 55.6 % in the post-period (*p* < 0.001).

**Conclusions:**

Our results indicate a statistically significant change in orthopedist prescribing patterns after guideline convergence. Furthermore, there was no apparent change in VTE risk between the two study groups when excluding patients necessitating full anticoagulation. Prior literature has shown that the divergence in guidelines influenced physicians away from ASA and toward more potent anticoagulants in order to avoid potential litigation. Once its role in VTEP was supported by the ACCP, it appears that ASA monotherapy was readily and rapidly incorporated into clinical practice. ASA may be favored over other VTEP agents for its lower bleeding risk profile and cost. This study highlights the profound impact clinical practice guidelines have on clinician prescribing patterns. Although prospective randomized trials are needed to compare the efficacy of ASA with other VTEP agents, ASA is now a predominant part of the VTEP armamentarium after KA.

## Background

Major orthopedic procedures confer increased risk of venous thromboembolic events (VTE), with rates reported as high as 60 % in the absence of chemoprophylaxis [1]. The use of chemoprophylactic agents has decreased the incidence of VTE following orthopedic procedures to 1–2 % [2, 3]. Despite significant improvement in outcomes, concern remains over VTE-related complications such as post-thrombotic syndrome, hemodynamic compromise from pulmonary embolism (PE), and death. Furthermore, VTE treatment itself presents risks including heparin-induced thrombocytopenia and major bleeding [4]. As rates of orthopedic procedures increase across the USA [5], the concern for VTE and its related complications grow. Between 1991 and 2010, annual primary total knee arthroplasty (TKA) volume increased 161.5 % from 93,230 to 243,802 [6]. The importance of venous thromboembolism prophylaxis (VTEP) becomes even more apparent when considering the financial burdens associated with VTE. Cost estimates range from $3000 to 9500 for the initial VTE, and those costs rise significantly when treating the sequelae [7].

Despite the importance of appropriate VTEP, until recently, medical and surgical professional societies presented divergent recommendations on choice of chemoprophylactic agent following joint replacement surgery. There remains limited evidence demonstrating efficacy and superiority of VTEP agents in extended use [8]. Additionally, the risk-benefit analysis balancing prevention of VTE with risk of major bleeding continues to contribute to a lack of consensus on VTEP regimens [8–10]. In 2009 and again in 2011, the American Academy of Orthopaedic Surgeons (AAOS) issued clinical practice guidelines (CPG) using a methodological approach with grades assessing the strength and evidence for patients undergoing hip or knee arthroplasty (KA). Their 2009 recommendations, for the first time, included aspirin (ASA) monotherapy as VTEP [11, 12]. Recommendations included ASA monotherapy as a chemoprophylactic agent at a dose of 325 mg twice-daily (BID) beginning on the day of surgery and continued for 6 weeks for patients without preoperative elevated VTE risk. Although the AAOS does not specify other VTE factors beyond previous VTE, the workgroup advised individualized assessment for patients deemed to be at elevated risk [13].

The 2011 AAOS recommendations gained support from the 2012 American College of Chest Physicians (ACCP) VTEP guidelines, which for the first time, included daily full-dose ASA (>300 mg) as acceptable chemoprophylactic monotherapy after total joint arthroplasty. The ACCP advised using ASA, low molecular weight heparin (LMWH), fondaparinux, apixaban, dabigatran, rivaroxaban, low-dose unfractionated heparin (LDUH), adjusted-dose vitamin K antagonist (VKA) (grade 1B recommendations; strong recommendation, moderate-quality evidence) for a minimum of 10–14 days following joint replacement surgery, with LMWH as the preferred agent (grade 2B recommendation; weak recommendation, moderate-quality evidence) [14, 15].

Evidence-based CPG are compiled to positively influence physicians’ practice [16–19]. CPG, including professional society guideline recommendations, have been shown to exert a powerful influence on providers and improve the quality of care administered to patients [16–23]. This investigation’s primary purpose is to determine whether the convergence of AAOS and ACCP CPG endorsing the inclusion of ASA monotherapy resulted in a significant change in orthopedic surgeon prescribing patterns.

## Methods

This is a retrospective IRB-approved collaborative cohort study between the Department of Orthopaedic Surgery and the Department of Internal Medicine. The study population consists of patients who underwent KA procedures at two tertiary care academic centers. We identified the patient sample using ICD-9 procedure codes (81.54, 00.80, 00.81, 00.82, 00.83, and 00.84) which were cross-referenced with ICD-9 diagnosis codes (715.16, 715.36, 996.49, 996.44, 715.96, 716.16, 715.35, 996.66, 714.4, 714.0, 714.9, 716.96, 736.5, and 996.77) to improve validity. The study period spanned July 1, 2008–July 31, 2014 with comparison periods of July 1, 2008–December 31, 2011 (pre-period) and January 1, 2012–July 31, 2014 (post-period). The data pool included every KA procedure performed by a total of 18 surgeons over a 7-year period. All data was obtained through the electronic health record (EHR). Included subjects were adults having a primary or revision KA. In an effort to control for patients that would necessitate more potent anticoagulation, we excluded patients who received preoperative full-dose anticoagulation and those with hypercoagulability. A biostatistician devised a systematic sampling algorithm to obtain a consistent number of patients for each year investigated and reduce bias that may arise due to single surgeon prescribing patterns. All chart reviewers underwent inter-reliability assessment to standardize the manner in which data was collected and cycled through the study years to eliminate the potential for systematic error. The study team constructed a comprehensive data dictionary to derive consensus on the variables examined.

Demographic data (age, gender, ethnicity, and insurance status) and validated VTE risk factors (medical co-morbidities, body mass index (BMI), personal history of VTE, estrogen therapy, history of malignancy, other surgery within three months, current tobacco use, and unilateral versus bilateral procedures) were collected in addition to the exclusion criteria [3, 24, 25]. We compiled data on all prescribed VTEP agents, including ASA 325 BID, warfarin sodium, LMWH, LDUH, apixaban, rivaraxiban, and fondaparinux, which were then aggregated into classes: ASA monotherapy, VKA, LWMH, Xa inhibitors, LDUH, and combination therapy. ASA monotherapy included ASA 325 BID or ASA 325 BID + clopidogrel; LMWH class included enoxaparin 30 or 40 mg/day, or 30 mg BID; VKA class included warfarin; Xa inhibitor class included fondaparinux, rivaroxaban or apixaban. Combination prophylaxis was defined as any combination of these agents. Time periods were chosen to reflect the introduction of ASA in the ACCP CPG. To assess changes in prescriptions during the hospital stay, we measured VTEP prescriptions on postoperative day 1 (POD1) and at time of discharge (D/C). The primary endpoint was the difference in rates of ASA monotherapy between the pre-period and post-period. Secondary endpoints were differences in chemoprophylactic agents prescribed on POD1 and at D/C, as well as associations between our adjusted VTE risk profiles and choice of agent.

The chi-square test was used to compare the rates of ASA VTEP between pre-period and post-period. Confidence intervals for the difference in rates were computed. In addition, exploratory graphical analysis was used to describe trajectories of ASA VTEP use over time. Smoothing techniques were used to determine whether trajectories obeyed a particular parametric pattern or patterns that could be modeled using multiple logistic regression as a function of time. Standard multiple logistic regression was used to estimate the probability of ASA VTEP in a specific patient as a function of the predictors listed above, as well as “era” (pre-2012, post-2012). The rates of VTEP change were compared across the two periods using the chi-square test for 2 × 4 tables. For patients who were not on ASA monotherapy, a descriptive list of prophylactic agents was produced.

## Results

**Fig. 1:**
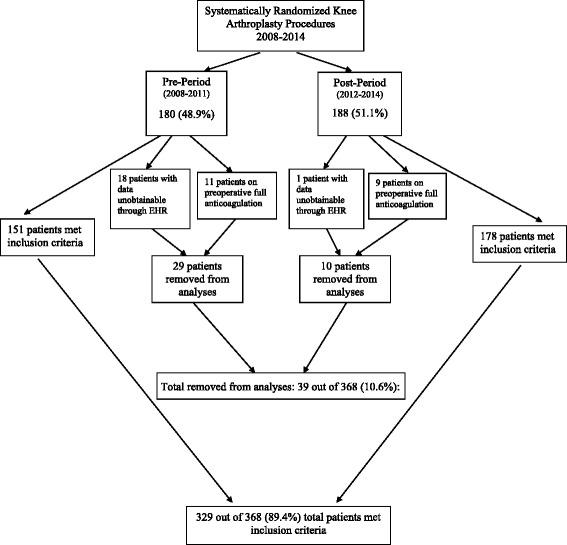
Study flow chart. A total of 329 patients were included in our study. This represents 89.4 % of patients originally selected via systematic randomization. Patients with a coagulopathy on preoperative anticoagulation or with data unobtainable through the EHR were removed

Of 368 records reviewed, 329 were included in the analysis. For the pre-period, there were 180 cases, 11 meeting exclusion criteria, and 18 with data unobtainable through EHR, resulting in 151 cases. For the post-period, there were 188 cases, 9 meeting exclusion criteria, and 1 with data unobtainable through EHR for a total of 178 cases (Fig. 1); 19 of the 368 charts (5.2 %) were irretrievable. In the pre-period, there were 55 male patients (36.4 %) with a mean age of 67.0 (±10.7) years. In the post-period, there were 67 male patients (37.6 %) with a mean age of 67.3 (±10.4) among all patients. The majority of patients in both the pre- and post-periods were obese (BMI >30) (Table 1).

**Table 1:** Patient characteristics

Variable	Pre-period	Post-period	*p* value
Male gender *n* (%)	55 (36.4)	67 (37.6)	NS
Age, years (standard deviation)	67.02 (10.73)	67.30 (10.42)	NS
BMI categories kg/m^2^			NS
Underweight <18.5 *n* (%)	0 (0)	1 (0.56)	
Normal weight 18.5–24.9 *n* (%)	12 (8.11)	13 (7.30)	
Overweight 25–29.9 *n* (%)	36 (24.32)	54 (30.34)	
Obese >30 *n* (%)	100 (67.57)	110 (61.80)	
Significant comorbidities			NS
None *n* (%)	141 (94.63)	166 (93.26)	
End-stage renal disease *n* (%)	2 (1.34)	0 (0)	
Coronary stents *n* (%)	5 (3.36)	12 (6.74)	
Cardiac valve replacement (%)	1 (0.67)	0 (0)	
Current smoker *n* (%)	22 (14.97)	27 (15.17)	NS
Malignancy history *n* (%)	21 (14.19)	25 (14.04)	NS
Estrogen therapy *n* (%)	1 (0.67)	2 (1.12)	NS
History of deep vein thrombosis *n* (%)	3 (2.01)	5 (2.81)	NS
Surgery in previous 3 months *n* (%)	2 (1.34)	1 (0.56)	NS
Bilateral TKA *n* (%)	5 (3.29)	2 (1.13)	NS

**Fig. 2:**
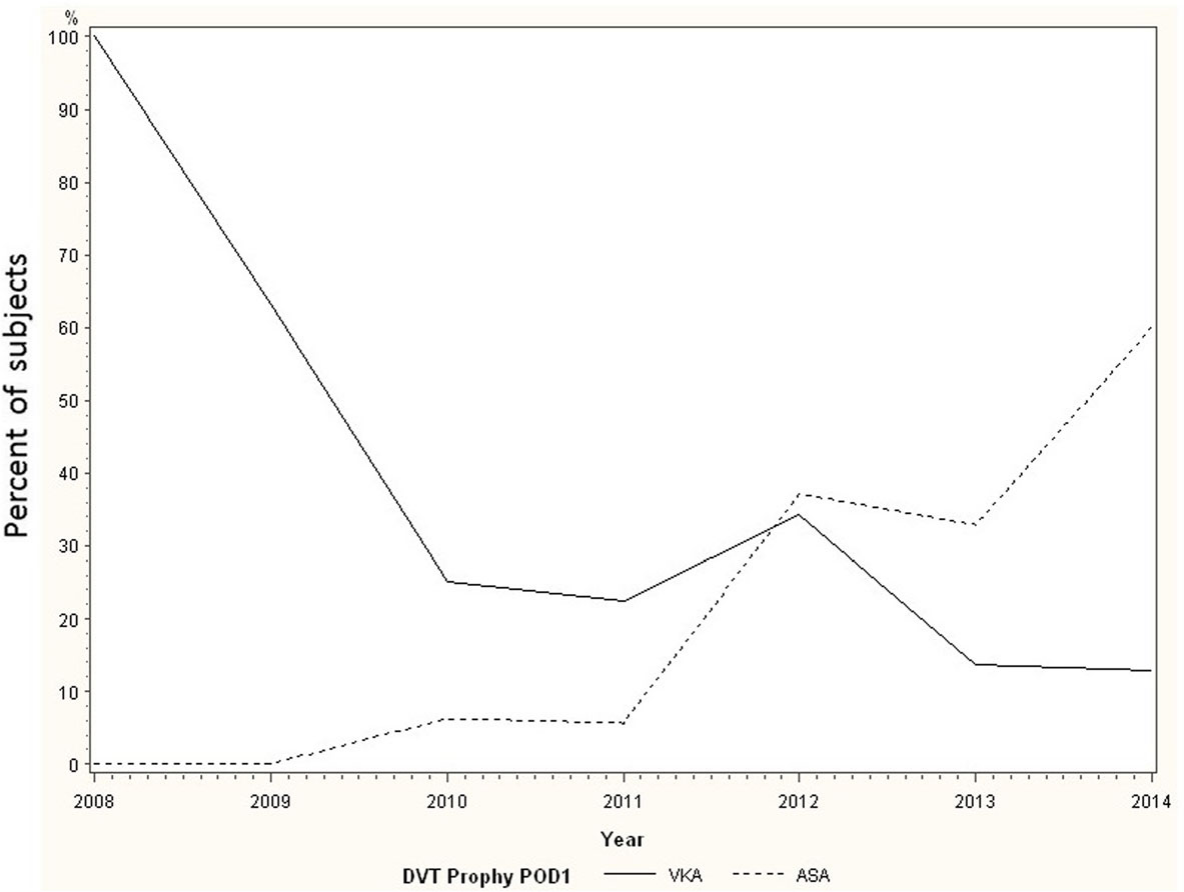
VTEP prescription rates on postoperative day 1. The percentage of patients prescribed ASA monotherapy on POD#1 increased significantly after guideline convergence. There was a simultaneous decrease in the percentage of patients prescribed VKA

**Fig. 3:**
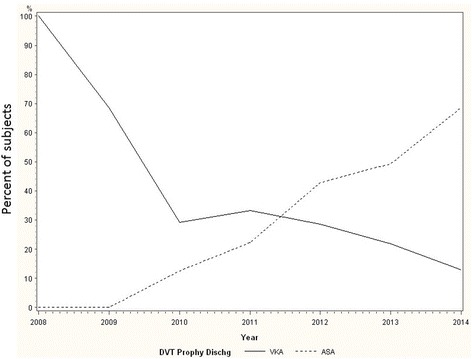
VTEP prescription rates on discharge. A similar change in VTEP prescribing was observed at discharge. A significant increase in ASA monotherapy prescribing occurred while VKA prescribing decreased

In the pre-period, 7/151 (4.6 %) subjects received ASA monotherapy on POD1 and 21/151 (13.9 %) received ASA monotherapy on D/C. In the post-period, 79/178 subjects (42.1 %) received ASA monotherapy on POD1 and 99/178 (57.8 %) received ASA monotherapy on D/C. For both POD1 and D/C, ASA monotherapy rates increased significantly from pre- to post-period (*p* < 0.0001) (Table 2). In the pre- and post-periods, a majority of subjects had 0–2 adjusted VTE risk factors (72.37 and 69.10 %, respectively). The proportion of subjects with three or more risk factors was also comparable between the pre- and post-periods (7.24 and 8.99 %, respectively). Overall, there was no statistically significant difference in the distribution of adjusted risk factors between the pre- and post-periods after excluding those patients that necessitate potent anticoagulation (Table 1). In the pre-period, the most commonly prescribed classes of agents were vitamin K antagonists (POD1 35.1 %, D/C 42.1 %) and Xa inhibitors (POD1 29.1 %, D/C 23.0 %). In the post-period, ASA monotherapy predominated at both time points (Table 2). There was a statistically significant inverse correlation between VKA and ASA monotherapy prescriptions (*p* < 0.0001 and *p* < 0.0001 for POD1 and D/C, respectively) during the study period (Figs. 2 and 3).

**Table 2:** Comparing prescribing patterns before and after guideline convergence

Class of VTEP agent	Pre-period POD1 *n* (%)	Post-period POD1 *n* (%)	*p* value	Pre-period discharge *n* (%)	Post-period discharge *n* (%)	*p* value
ASA monotherapy^a^	7 (4.64)	79 (44.38)	<0.0001	21 (13.91)	99 (55.62)	<0.0001
All other agents	144 (95.36)	99 (55.62)		130 (86.09)	79 (44.38)	
LMWH^b^	32 (21.19)	10 (5.62)		26 (17.22)	13 (7.30)	
Vitamin K antagonist	57 (37.75)	31 (17.42)		64 (42.38)	35 (19.66)	
Xa inhibitors^c^	44 (29.14)	36 (20.22)		35 (23.18)	20 (11.24)	
Combination^d^	11 (7.28)	20 (11.24)		5 (3.31)	11 (6.18)	
LDUH 5000 U TID	0 (0)	2 (1.12)		0 (0)	0 (0)	

^a^Includes ASA325 BID, ASA325 BID + clopidogrel

^b^Includes enoxaparin 40 mg/day, enoxaparin 30 mg/day, enoxaparin 30 mg BID

^c^Includes fondaparinux, rivaroxaban, apixaban

^d^Includes Xa inhibitor + ASA81, warfarin + Xa inhibitor, warfarin + ASA81, warfarin + clopidogrel, warfarin + ASA325 + clopidogrel, ASA325BID + Xa inhibitor, warfarin + enoxaparin, ASA325 + enoxaparin

## Discussion

Patients undergoing lower extremity orthopedic procedures remain at high risk for developing VTE [1–3]. Orthopedic surgeons are dedicated to reducing this risk, while preventing adverse side effects associated with certain anticoagulant agents [13]. Conflicting CPG and a paucity of high-level evidence have contributed to clinician confusion regarding VTEP decision-making. In 2012, two prominent professional organizations moved toward closer alignment by including ASA monotherapy as an acceptable chemoprophylactic agent [13, 15]. The results of our study show that there was a statistically significant increase in ASA monotherapy prescriptions after the convergence of AAOS and ACCP CPG, thus supporting the notion that CPG can influence physician practices.

The results of our study can help other clinicians overcome what has been described as “inertia of previous practice” [26], provide guidance on VTEP agent selection, and ultimately highlight the profound impact of CPG on clinician prescribing patterns. While some authors suggest that CPG may have a limited impact, other literature on the role of CPG suggests that nationally developed guidelines, especially when endorsed by professional specialty organizations, are a key element to altering physician behavior [16–18, 20, 27–32]. CPG appear to play a large role in VTEP selection, especially when considering that in the absence of an obvious optimal agent, many surgeons state that they rely on guidelines put forth by leading specialty organizations for practice guidance [31]. Furthermore, there is an increased acceptance of national guidelines when colleagues endorse change and incorporate it into practice [33, 34].

Prior to 2012, orthopedic surgeons were aware of VTEP CPG and data supporting the use of ASA but hesitated to adopt new practices. A 2008 survey of the members of the American Association of Hip and Knee Surgeons (AAHKS) suggested that roughly 90 % of respondents were familiar with the AAOS or ACCP guidelines. The AAHKS survey provided key perspectives on the then divergent AAOS and ACCP guidelines: 82 % of surgeons agreed more with the AAOS guidelines and 74 % believed the ACCP guidelines were not relevant to orthopedics. Although respondents agreed that enoxaparin was the most efficacious agent, 68 % of surgeons reported that ASA was the easiest to use with the lowest risk profile for bleeding and wound drainage [31].

The AAHKS survey provided key insight regarding VTEP agent selection. The divergence in guidelines steered physicians away from ASA and toward the more potent anticoagulants, such as LMWH or VKA, advocated by the ACCP. The same survey revealed that 53 % of surgeons had adjusted their practice based on guidelines issued by AAOS and ACCP, with a significant portion acknowledging that malpractice claims had directly influenced a change in their practice [31]. Despite data supporting the use of ASA, orthopedic surgeons had resisted changing their prescribing patterns for malpractice defensive concerns [26, 35].

Numerous possible factors may be responsible for our study conclusion. In one recent study, potent anticoagulants such as LMWH, fondaparinux, or VKA were associated with higher all-cause mortality and incidence of clinical non-fatal PE after hip and KA [26]. Additionally, data suggests that ASA is comparable to other agents in preventing VTE and non-fatal PE [10, 36]. Moreover, the pulmonary embolism prevention trial demonstrated a protective effect of ASA against symptomatic deep vein thrombosis (DVT) and PE of 29 and 43 %, respectively, versus placebo [9]. Perhaps paramount of all, ASA may be favored clinically for its lower bleeding risk profile [10, 37, 38] and its rapid, inexpensive reversal capability [39]. These benefits make ASA favorable for concomitant use in regional anesthesia as opposed to other agents, such as LMWH, where it is a relative contraindication [10]. Finally, ASA is cost-effective, as it does not require hematologic monitoring or insurance approval [8]. The capability of ASA as a VTEP agent in “at risk” populations is documented in both the medical and orthopedic literature [9, 10, 36]. Prior to guideline convergence, orthopedic surgeons were admittedly influenced by the legal ramifications of their prescribing patterns and generally opted for more potent agents with less optimal side effect profiles.

While evidence supporting the increased risk of more potent anticoagulants became available during or after 2014 [40–42], surgeons were already cognizant of the increased risk associated with more potent anticoagulants. This discrepancy may confound our conclusion and help explain the gradual shift toward ASA seen over the entirety of the study period. However, there was a statistically significant increase in ASA monotherapy prescriptions after the convergence of ACCP and AAOS recommendations.

**Fig. 4:**
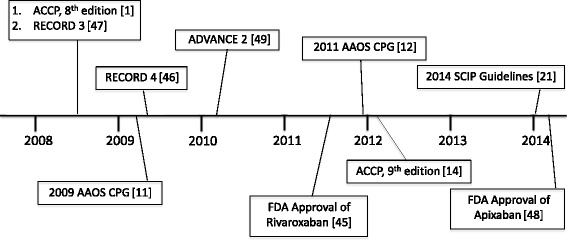
Timeline of relevant VTEP events. Significant events related to VTEP prescribing after TKA during our study period. Events related to CPG, relevant randomized clinical trials, and FDA approval of medications were included

Vitamin K antagonists and Xa inhibitors were the most widely prescribed VTEP agents in the pre-period, while ASA predominated post-convergence. The observed use of Xa inhibitors is noteworthy due to the timing of their development and approval (Fig. 4). The use of Xa inhibitors for VTEP after KA was supported by the AAOS and ACCP prior to their convergence on ASA [1, 11, 12, 15]. The FDA approved fondaparinux in 2001 for VTEP after hip and knee replacement surgery [43]. A randomized clinical trial showed fondaparinux to be more effective in preventing VTE than enoxaparin (30 U BID) in patients undergoing elective major knee surgery, but with an increased risk of major bleeding [44]. The FDA approved rivaroxaban in 2011 for VTEP after hip and knee replacement surgery [45]. The RECORD 3 and RECORD 4 randomized trials showed rivaroxaban to be superior to enoxaparin for VTEP after TKA, with similar bleeding rates [46, 47]. The FDA approved apixaban for VTEP after hip and knee surgery in 2014 [48]. The ADVANCE-2 randomized clinical trial showed apixaban to be a convenient and more effective alternative to Lovenox after KA without increased bleeding risk [49]. However, the lack of an available Xa inhibitor reversal agent is a major concern for clinicians [50]. Although the 2008 AAHKS member survey respondents felt that fondaparinux was more effective than ASA, the perceived bleeding risk and lack of reversal agent steered respondents away from Xa inhibitors [31]. This disadvantage of Xa inhibitors further elucidates the appeal of ASA monotherapy for VTEP after KA.

Various factors impact providers’ VTEP agent choice. Assessing VTE risk is multifactorial and often requires an individualized patient-specific approach. There were no differences in preoperative VTE risk between our two study groups (after excluding patients that necessitated full anticoagulation), showing that there is no bias toward more potent anticoagulation in one study population versus the other. In 2014, the ACCP published a weighted risk index utilizing seven VTE risk factors in order to aid providers in risk-stratifying surgical patients. The risk model analyzed the following factors: history of VTE, current neoplasm, sepsis, age greater or equal to 60 years, BMI greater or equal to 40 kg/m^2^, male sex, and family history of VTE [3]. We examined five of these factors with other validated factors associated with increased VTE risk (estrogen therapy [24], smoking status, unilateral versus bilateral procedures [25], or surgery within 3 months). Family history of VTE was not available from the EHR and thus not included. None of the patients in our study had a diagnosis of sepsis immediately prior to surgery.

Financial implications influence prescribing patterns as well [51]. As such, one potential confounder to our conclusion is the inclusion of ASA in the new Surgical Care Improvement Project (SCIP) recommendations [21]. Adherence to core measures, such as SCIP, is directly related to reimbursement. In 2014, the Joint Commission amended the recommendation for VTE prophylaxis for SCIP to include aspirin as an acceptable agent. However, the differences in ASA prescribing seen in our study were significant prior to these 2014 changes.

Although prospective randomized trials are needed to compare the efficacy of ASA with other VTEP agents, ASA is now a predominant part of the VTEP armamentarium after KA. Although only two institutions were reviewed, we believe that our results can be representative of medical practice in the USA because of the diversity of our patient population. Limitations include the retrospective design, which may diminish the validity of the study conclusion. Also, retrieval analysis limitations have the potential to introduce selection and/or transfer bias; 19/368 charts (5.2 %) were unable to be retrieved.

## Conclusions

With no difference seen in the adjusted VTE risk between the two study populations, we conclude that the convergence of AAOS and ACCP CPG influenced orthopedic surgeons to incorporate ASA monotherapy into clinical practice for VTEP after KA procedures.

## Abbreviations

ACCP: American College of Chest Physicians
BID: Twice-daily
CPG: Clinical practice guidelines
D/C: Discharge
EHR: Electronic health record
KA: Knee arthroplasty
LDUH: Low-dose unfractionated heparin
LMWH: Low molecular weight heparin
POD1: Post-operative day 1
VKA: Vitamin K antagonist
VTE: Venous thromboembolism
VTEP: Venous thromboembolism prophylaxis
